# Verisyse versus Veriflex Phakic Intraocular Lenses: Refractive Outcomes and Endothelial Cell Density 5 Years after Surgery

**DOI:** 10.1155/2018/4210460

**Published:** 2018-09-30

**Authors:** Dilek Yaşa, Alper Ağca

**Affiliations:** Beyoğlu Eye Research and Training Hospital, Bereketzade Mah, No. 2, Beyoglu, Istanbul, Turkey

## Abstract

**Purpose:**

To compare refractive stability, central endothelial cell density (ECD), and complications between Verisyse (Abbott Medical Optics, Netherlands) and Veriflex (Abbott Medical Optics, Netherlands) phakic intraocular lenses (pIOL) over five years.

**Methods:**

We retrospectively reviewed the medical records of patients who underwent Verisyse or Veriflex pIOL implantation for surgical correction of myopia. Patients with a 5-year follow-up period were included in the study. Uncorrected distance visual acuity (UDVA), corrected distance visual acuity (CDVA), spherical equivalent of manifest refraction (SE), and ECD were compared between the groups preoperatively and 1, 3, and 5 years postoperatively.

**Results:**

The study included 47 eyes in the Verisyse group and 50 eyes in the Veriflex group. There was no significant difference in mean SE, UDVA, CDVA, and ECD preoperatively or postoperatively. In both groups, there was a statistically significant myopic shift between 1-year and 5-year visits (−0.25 ± 0.30 D and −0.23 ± 0.48 D in the Verisyse and Veriflex groups, respectively). There was no significant difference between the groups in terms of efficacy and safety indexes at 5 years. ECD loss was highest during the first year (3.9% loss in the Verisyse group and 3.9% loss in the Veriflex group, *p*=0.670). At 5 years, the mean cumulative ECD losses in the Verisyse and Veriflex groups were 7.42% and 7.64%, respectively (*p*=0.709). Cataracts developed in 2.1% of the eyes in the Verisyse group and in 2.0% of those in the Veriflex group. No sight-threatening complications were observed.

**Conclusion:**

Verisyse and Veriflex pIOLs are highly effective for treating high myopia up to 5 years after surgery. Longitudinal studies with longer follow-up periods are necessary to determine the endothelial safety profile.

## 1. Introduction

Phakic intraocular lens (pIOL) implantation is a surgical method for treatment of high myopia [1]. Other options are corneal refractive surgery and clear lens extraction [2, 3]. pIOL implantation offers some advantages in highly myopic individuals. It allows maintenance of accommodation and results in better quality of vision when compared with corneal refractive surgery [4].

However, most early designs have been abandoned because of high rates of complications such as cataracts, glaucoma, and excessive endothelial cell loss in the long term [5, 6]. Verisyse (Abbott Medical Optics, Netherlands) and Veriflex (Abbott Medical Optics, Netherlands) are pIOLs that are considered to have good safety and efficacy [7–10]. Both of them are implanted in the same location with the same mechanism of fixation, but they have different material properties and require different incision sizes. Thus, they may have different efficacy and safety profiles.

There are only a limited number of studies comparing the long-term clinical outcomes following implantation of these lenses [11]. As a result, there is still a need for data on long-term follow-up and comparison of these lenses to establish their long-term efficacy and safety profiles. The aim of this study was to compare Verisyse and Veriflex in terms of the refractive results, central endothelial cell density (ECD), and complications over the long term.

## 2. Materials and Methods

This study was approved by the Institutional Review Board. Tenets of Declaration of Helsinki were followed. Medical records of patients who underwent an iris-claw pIOL implantation were evaluated retrospectively. The operative records of a single surgeon (senior author, AA) were queried, and patients with a 5-year follow-up period were included in the study. Uncorrected distance visual acuity (UDVA), corrected distance visual acuity (CDVA), spherical equivalent of manifest refraction (SE), and ECD were compared between the groups preoperatively and 1, 3, and 5 years postoperatively.

All patients received a full ophthalmological examination including refraction, UDVA and CDVA measurement, slit-lamp evaluation, Goldman applanation tonometry, fundoscopy, anterior-chamber depth measurement (from endothelium) using IOL Master (Carl Zeiss Meditec, Germany), and ECD measurement using a specular microscope (CEM 530, NIDEK, Japan). The patients were scheduled for yearly follow-up after the first year of surgery, which is routine in our clinic. A lens opacity that results in the loss of ≥2 lines of CDVA during follow-up was defined as cataract.

### 2.1. Verisyse Phakic Intraocular Lens Implantation

Myopic model 206 was used for myopia less than −15.5 D, and model 204 was used for higher myopia. The target was emmetropia in all cases. Power calculation was performed using the modified vergence formula provided by the manufacturer. A surgical caliper was used to mark the planned borders of a 6 mm main incision centered at 12 o'clock. Two paracenteses were performed on two sides of the planned main incision. Acetylcholine 0.01% (Miochol-EO, Novartis) was injected into the anterior chamber from one of the paracenteses to constrict the papilla. The anterior chamber was filled with a cohesive viscoelastic material (Provisc, Alcon), and then the main incision was performed. The Artisan IOL was introduced from the main incision and rotated inside the eye until it was horizontal. The IOL optic was grasped with specially designed forceps, and the iris was enclaved in the claws of the pIOL using a special needle introduced from the paracentesis. Iridotomy was performed, and two interrupted nylon sutures were used to close the main incision.

### 2.2. Veriflex Phakic Intraocular Lens Implantation

The target was emmetropia in all cases. Power calculation was performed using the modified vergence formula provided by the manufacturer. Two paracenteses were performed on two sides of the planned main incision. Next, 0.01% acetylcholine (Miochol-EO, Novartis) was injected into the anterior chamber from one of the paracentheses to constrict the pupilla. A 2.75 mm main incision centered at 12 o'clock was performed with a slit knife. The anterior chamber was filled with a cohesive viscoelastic material (Provisc, Alcon), and the Veriflex IOL was introduced from the main incision and rotated inside the eye until it was horizontal. The IOL optic was grasped with specially designed forceps, and the iris was enclaved in the claws of the pIOL using a special needle introduced from the paracentesis. Iridotomy was performed, and the incisions were hydrated with BSS.

### 2.3. Statistical Methods

Statistical analysis was performed using SPSS for Windows (version 21.0; IBM, Armonk, NY), and the associated graphics were generated with Microsoft Excel 2013 (Microsoft Corporation, Seattle, WA, USA). The mean, standard deviation, and frequency were used in the statistical analysis. The variable distribution was determined using the Shapiro–Wilk test. A paired *t*-test was used to analyze parametric data, and a Wilcoxon signed-rank test was used to analyze nonparametric data. Chi-square and Fisher's exact tests were used to compare categorical variable. One-way repeated measures analysis of variance (ANOVA) was used to evaluate ECD during follow-up. The annual ECD loss was calculated according to the following formula:

(1) $$\begin{array}{c} \text{ECD loss}/\text{year}=\frac{\text{ECDf}-\text{ECDi}}{\text{ECDi}\times t}, \end{array}$$

where ECDf is the endothelial cell count at the last visit, ECDi is the preoperative cell count, and *t* is the time in years between the two endothelial cell count measurements.

## 3. Results

The study included 97 eyes from 63 subjects. There were 40 (63%) male subjects and 23 (37%) female subjects. There were 47 eyes in the Verisyse group and 50 eyes in the Veriflex group. The preoperative patient characteristics are shown in Table 1. There were no statistically significant differences between the preoperative characteristics of the groups.

**Figure 1:**
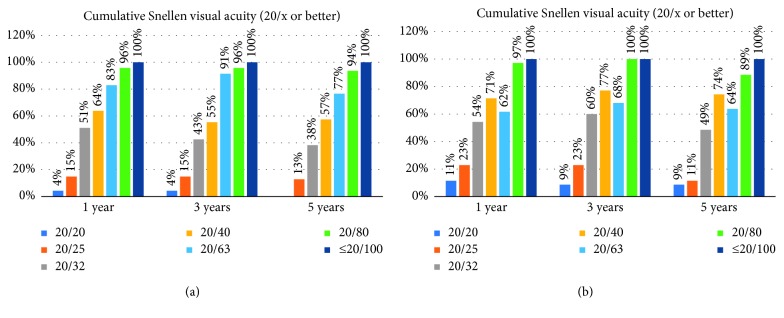
Cumulative uncorrected distance visual acuity in Verisyse (a) and Veriflex (b) groups.

Figures 1(a) and 1(b) show the postoperative cumulative Snellen visual acuities (UDVA and CDVA) of the Verisyse and Veriflex groups, respectively. The efficacy indices (preoperative CDVA and postoperative UDVA) at 5 years were 1.14 ± 0.60 and 1.04 ± 0.47 in the Verisyse and Veriflex groups, respectively (*p* > 0.05).

Table 2 shows the SE of manifest refraction preoperatively and at postoperative visits. In both groups, SE was similar between the groups preoperatively and at the postoperative visits at 1, 3, and 5 years. However, postoperative SE increased significantly during the five-year follow-up in both groups. In both groups, there was a statistically significant myopic shift (−0.25 ± 0.30 D and −0.23 ± 0.48 D in Veriflex and Verisyse groups, respectively) between 1-year and 5-year visits. At the end of the follow-up (5-years), the mean SE was −0.68 ± 0.44 and −0.72 ± 0.40 in the Veriflex and Verisyse groups, respectively. Tables 3 and 4 list refractive sphere and refractive cylinder during follow-up. The refractive sphere increased significantly during the five-year follow-up in both groups. In Veriflex group, there was no significant change in the refractive cylinder during follow-up. In Verisyse group, the refractive cylinder was not significantly different at the end of follow-up when compared with preoperative cylinder.

**Figure 2:**
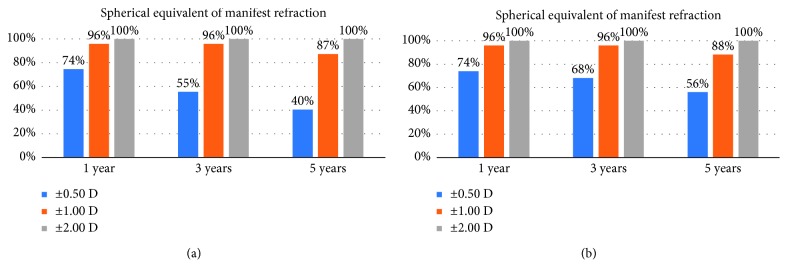
Spherical equivalent of mean manifest refraction in Verisyse (a) and Veriflex (b) groups.

At the 1-year postoperative visit, 74% of the eyes in both groups were within ±0.5 D of emmetropia. However, at the 5-year postoperative visit, only 40% and 56% of the eyes were within ±0.5 D of emmetropia in the Verisyse and Veriflex groups, respectively (Figures 2(a) and 2(b)). The difference between the groups was statistically insignificant (Chi-square test, *p*=0.156).

**Figure 3:**
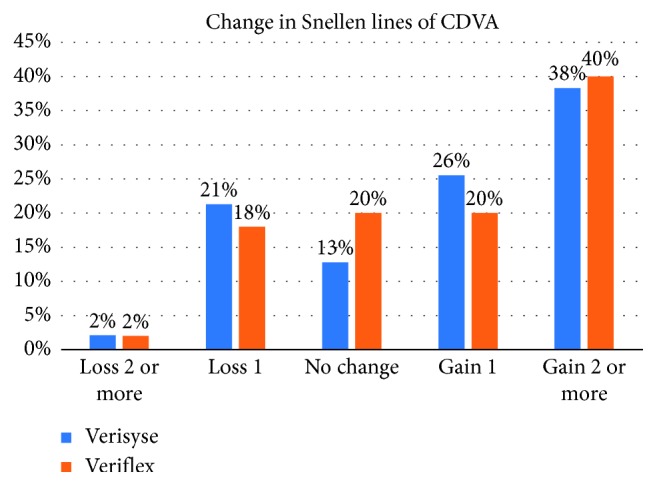
Change in corrected distance visual acuity (CDVA).

None of the patients lost ≥2 lines of CDVA (Figure 3). The safety indices (preoperative CDVA and postoperative CDVA) at 5 years were 1.39 ± 0.63 and 1.31 ± 0.50 in the Verisyse and Veriflex groups, respectively (*p* > 0.05).

In one patient in the Veriflex group, one of the haptics of the iris-claw lens was refixated at the 1-year visit because it was loosely fixated to the iris. It was mobile and slightly decentered. This patient did not report a history of trauma, allergy, or eye-rubbing behavior, and the pIOL was stable and centralized at the last follow-up. The decentration was probably due to inappropriate enclavation during the surgery. The haptic was re-enclaved with a second operation just after the 1-year visit, and the patient experienced no further complications. Intraocular pressures (IOP) during follow-up are listed in Table 5. In all patients, IOP was ≤21 mmHg at all postoperative visits.

Table 6 shows the endothelial changes over the course of the study. ECD was similar between the groups preoperatively and postoperatively at all visits. At 5 years, the mean cumulative ECD losses in the Verisyse and Veriflex groups were 7.42% and 7.64%, respectively (*p*=0.709). None of the patients lost ≥25% of their baseline ECD during the 5-year follow-up. Annual ECD loss for Verisyse was 3.05% in the first year, 1.23% between 1 and 3 years, and 1.02% between 3 and 5 years. The annual ECD loss for Veriflex was 3.05% in the first year, 1.24% between 1 and 3 years, and 1.05% between 3 and 5 years.

## 4. Discussion

In this study, we evaluated and compared the long-term results after implantation of two different types of iris-claw pIOLs. In line with previous studies, the refractive and visual results were satisfactory in both groups. There were no significant differences between the groups in terms of MRSE, UDVA, and CDVA during the follow-up period.

There are only a few studies comparing these two pIOLs in the long term. Bohac et al. [11] compared the refractive outcomes of Verisyse and Veriflex pIOLs for 36 months after surgery and found that SE was similar at the 36-month visit. However, CDVA was significantly better in the Veriflex group, in contrast to our study. An improvement in CDVA has been reported in the literature, and results from the magnification effect of pIOLs were compared with spectacles. A difference between the CDVA of the groups is difficult to reveal before the operation, and the difference probably results from preoperative patient characteristics in the study reported by Bohac et al. [11].

High refractive predictability after phakic iris-claw has been reported [7–9,11,12]. In a clinical trial study from the United States Food and Drug Administration (FDA), Stulting et al. reported that 71.7% of eyes were within 0.5 D of the target refraction and 94.7% were within 1.0 D postoperatively. In line with the FDA study, we found that 74% and 96% of the eyes in the Verisyse group were within ±0.5 D and ±1.00 D, respectively [7]. The percentages of the eyes that were in ±0.5 D and ±1.00 D were equal in the Veriflex group. However, we found that the refractive results were not stable in the long term. There was a small but statistically significant regression at the 1-year and 5-year visits in both groups (−0.25 ± 0.30 D and −0.23 ± 0.48 D in the Verisyse and Veriflex groups, respectively). The amount of regression was not statistically different in both groups, and it is probably related to an increase in axial length. Several studies report that the refractive results are stable after pIOL implantation, while others report that regression of the refractive effect occurs, and it is probably related to a progressive increase in the axial length in at least some patients. Guell et al. [13] reported a 5-year follow-up study of 399 phakic Artisan-Verisyse IOLs. Among the patients with myopia, only 10% of the eyes in the 5 mm optic Verisyse PIOL group and 38% of the eyes in the 6 mm optic Verisyse PIOL group were within 0.5 D of the target refraction. Additional refractive surgery was performed in 60% and 20% of the 5 and 6 mm optics groups, respectively. The stability in the refractive results in several studies probably results from the amount of initial myopia, relatively short follow-up, low number of patients, or lack of sufficient statistical power to find a difference, or a combination of these factors.

Despite a regression during follow-up, we found the efficacy index to be 1.14 and 1.04 in the Verisyse and Veriflex groups (*p* > 0.05), respectively. It is also our clinical experience that most patients still have a UDVA equal to or better than the preoperative CDVA, even in the presence of a residual refractive error. The increase in CDVA is probably the reason for the high efficacy index, despite the residual refractive errors and a small but statistically significant regression over time.

Approximately 20% of the eyes gained ≥1 line, and one-third of the eyes gained ≥2 lines. The exact mechanism of the increase in CDVA is unclear, but the reason for the improvement may be the relative magnification of the image after an anterior chamber pIOL implantation when compared with spectacle lenses [14]. There is agreement in the literature that improvement occurs in CDVA after corneal refractive surgery or pIOL implantation [15–17].

A prospective, multicenter U.S. FDA trial reported the most detailed data with the highest level of evidence on the ECD loss after a similar rigid iris-claw pIOL (Artisan). The ECD loss was −4.8% ± 7.8% from baseline to 3 years, with a 2.4% loss between 2 and 3 years [7]. Benedetti et al. [18] reported an ECD loss of 4.7% at two years and 9% at five years after Artisan pIOL implantation. A European prospective multicenter study evaluated the surgical results of Artiflex, and the mean endothelial cell changes were -0.05%, 1.79%, and -1.07% at 6 months, 1 year, and 2 years, respectively [10]. All of these values are higher than the normal ECD loss in a nonoperated eye. The ECD loss after implantation of iris-claw and posterior chamber phakic IOLs are comparable. Thus, it is not clear if one design has an advantage over the other in terms of ECD [7,18–20]. The most detailed data with the highest level of evidence on the ECD loss after a posterior chamber pIOL implantation was reported during the U.S. FDA trial (526 eyes, 4 years). In that study, ECD loss was 3.3 ± 7.6% at one year (90% confidence limits: 2.4% to 4.3%) and 9.7 ± 9.3 at 4 years [19, 20]. Recently, Yasa et al. [21] reported a mean ECD loss of 3.9% at one year after implantation of a new posterior chamber phakic IOL.

Several studies report that endothelial damage occurs primarily during the surgical procedure, and the decrease stabilizes after a certain time [22–24]. However, there are reports of late endothelial decompensation, which indicate progressive cell loss in at least some patients. Saxena et al. [25] found a significant negative correlation between ACD and endothelium cell loss. Doors et al. [26] found that a shallow ACD and smaller distance between the pIOL edge and the endothelium were risk factors for ECD loss. In a recent study, Jonker et al. [27] reported that in a group of 507 eyes, ECD had decreased by ≥25% in 7.9% of them, and ECD was <1500 cells/mm^2^ in 3.9% at 10 years after implantation. Risk factors for increased ECD loss were a shallow ACD and a smaller distance between the central and peripheral pIOL edge to the endothelium. However, it is still not clear if ECD loss in eyes that do not have these risk factors is still higher than normal ECD loss in a nonoperated eye.

Verisyse and Veriflex have similar designs and fixation mechanisms but different material properties. Hedayatfar et al. [28] compared chronic subclinical inflammation induced after implantation of Artisan versus Artiflex phakic intraocular lenses (pIOLs). Anterior chamber flare was assessed quantitatively using laser flare photometry (LFP). They concluded that the inflammatory response induced by implantation of either type of pIOLs is short-lived without statistically significant difference between the two models. However, there are reports showing that the silicon optic material that is used in Veriflex may cause inflammation and might be increasing the incidence of pigment deposits postoperatively [10, 29, 30]. No chronic inflammation was seen in our patients. However, inflammation was not assessed quantitatively, and we highlight the possibility that mild inflammation in the early postoperative period could have gone unnoticed. This weakness of the study should be considered when interpreting the results.

Although there are several studies reporting long-term ECD loss for both phakic IOLs, it is difficult to compare the effect of lenses on ECD. It is difficult to draw a conclusion by comparing different studies because surgeries are performed by different surgeons and preoperative patient characteristics are different in these studies. For example, in the prospective multicenter U.S. FDA trial, one site had a mean cell loss of 5.0%, and the others combined had a mean cell loss of 1.7% [13].

In this study, we evaluated two different iris-claw lenses with different material properties. All the surgeries were performed by a single surgeon, all the patients had ACD ≥ 3.00 mm from the endothelium, and preoperative patient characteristics were similar between the groups. In addition, the follow-up duration was reasonably long. We found that the central ECD loss was similar in both groups at all follow-up points. ECD loss was highest during the first year (3.05% and 3.04% in the Verisyse and Veriflex groups, respectively), and at five years, the cumulative loss was 7.42% and 7.64% in the Verisyse and Veriflex groups, respectively. The results correspond to an annual ECD loss of 1.02 and 1.05% in Verisyse and Veriflex pIOLs between 3-year and 5-year visits.

ECD loss was highest during the first year and diminished thereafter. However, it is still not possible to conclude that the ECD loss is similar to a normal nonoperated eye. In early cross-sectional studies, the average annual endothelial cell loss rate in normal eyes was found to be approximately 0.3 to 0.5% [31, 32]. In a longitudinal study, in which the same subjects were examined again at a later date, Bourne et al. reported an annual loss of 0.6 ± 0.5% over 10 years [33]. In a recent longitudinal study, the annual rate of cell loss after refractive surgery was found to be 0.6% ± 0.8 % over 9 years [34]. Thus, we believe that life-long ECD follow-up is needed in patients who have undergone pIOL implantation.

In both groups, there were no serious intraoperative complications, and the safety index was not statistically significantly different between the groups. None of the patients lost more than 2 lines, and 1 patient in both groups lost 2 lines at 5 years (2.1% and 2.0% in the Verisyse and Veriflex groups, respectively). In both patients, the reason for CDVA loss was cataracts. Most of the patients undergoing pIOL implantation are young adults. Thus, cataract formation is a major concern when implanting a pIOL. In a meta-analysis of 6,338 eyes, Chen et al. [35] reported that the incidence of cataract formation was 1.29%, 1.11%, and 9.60% with anterior-chamber, iris-fixated, and posterior-chamber pIOLs. However, the rate of cataract formation may be higher in longer follow-up. Moshirfar et al. [36] evaluated the incidence rate and indications to investigate Verisyse pIOLs implanted over a 13.6-year period by one surgeon at one institution with a mean follow-up of 5.6 years per eye. Similar to our study, they reported that the occurrence of cataract formation in this patient population was 2.3%.

In our patient group, the only other complication was a slight decentration of the pIOL due to improper enclavation of one of the haptics in the iris. This patient did not report a history of trauma, allergy, or eye rubbing, and the decentration was probably due to inappropriate enclavation during the surgery. The haptic was re-enclaved with a second operation just after the 1-year visit, and the patient did not have any additional complications in the remaining follow-up period. No pigment dispersion, glaucoma, or pupillary block was observed in this patient group.

The most important weakness of this study is its retrospective nature. For example, there were no cases of pigment dispersion in our patients. Although intraocular pressure measurement is routine at every visit in our clinic, gonioscopy was not performed. Thus, very mild clinical pigment dispersion in some patients could have gone unnoticed.

We found a myopic shift during 5 years in our patient group. This was an expected finding as most of our patients had high axial myopia. Thus, a correlation could have been found between the axial length (AL) change and the change in SE if we had performed an analysis. However, it is unusual to measure AL routinely in postoperative visits. Thus, AL measurement was not part of our routine postoperative examinations. Accordingly, neither this study nor the other retrospective studies in the literature report an analysis of correlation between the change in SE and the change in AL [18, 22, 25]. This is a weakness of this study that results from its retrospective nature and can be addressed in prospective studies.

Postoperative examinations were performed by different residents during the 5-year follow-up, and mild anterior subcapsular cataracts that do not affect visual acuity or slight pupil ovalization in a patient may have gone unnoticed. In addition, it was not possible to measure the distance between the pIOL edge and the endothelium postoperatively to evaluate its long-term effect on ECD because of the retrospective nature of the study. However, the advantages of this study are the longitudinal follow-up of ECD measurements for 5-years in 97 patients for two different pIOLs implanted by the same surgeon in patients with similar preoperative characteristics and a minimum ACD of 3.02 mm.

In conclusion, we have found that refractive results and visual acuities were clinically similar after implantation of both designs of iris-claw pIOLs in patients with high myopia. Both pIOLs were highly effective for the surgical treatment of high myopia, and the incidence of perioperative and postoperative complications is rare when patients are selected carefully. Central ECD loss was similar in both the Verisyse and Veriflex groups and slowed down dramatically after the first year. However, we believe that ECD and intraocular lens position should continue to be monitored as these patients are usually young and will continue to live with the implanted lens for many decades. Prospective studies with larger patient groups and longer follow-up periods are needed to reveal long-term ECD loss profiles.

## Figures and Tables

**Table 1:** Preoperative characteristics.

Parameter	Verisyse group	Veriflex group	*p*
	Mean ± SD (range)	Mean ± SD (range)	
Age (years)	31 ± 5 (20 to 42)	30 ± 5 (20 to 41)	0.247
SE (D)	−12.50 ± 3.51 (−6.25 to −20.00)	−11.50 ± 3.46 (−4.75 to −20.75)	0.207
Cylinder (D)	0.75 ± 0.53 (0 to 2.00)	0.59 ± 0.56 (0 to 2.00)	0.176
CDVA (logMAR)	0.34 ± 0.22 (0 to 1.00)	0.26 ± 0.16 (0 to 0.70)	0.095
ECD (cells/mm^2^)	2681 ± 275 (2278 to 3220)	2656 ± 270 (2258 to 3205)	0.692
ACD (mm)	3.27 ± 0.21 (3.03 to 3.69)	3,32 ± 0.26 (3.02 to 3.82)	0.316
AL (mm)	28.44 ± 1.58 (24.45 to 31.62)	28.11 ± 1.49 (23.70 to 30.08)	0.342

SD: standard deviation; D: diopters; SE: spherical equivalent; CDVA: corrected distance visual acuity; WTW: white-to-white; ECD: endothelial cell density; Sim K: simulated keratometry; IOP: intraocular pressure; AL: axial length.

**Table 2:** Spherical equivalent (SE) of subjective manifest refraction during follow-up.

	Preoperative (mean ± SD)	1 year (mean ± SD)	3 years (mean ± SD)	5 years (mean ± SD)	*p*
Veriflex	−11.50 ± 3.46	−0.36 ± 0.43	−0.49 ± 35	−0.68 ± 0.44	*p* < 0.001^*∗*^
Verisyse	−12.50 ± 3.51	−0.43 ± 0.34	−0.54 ± 0.39	−0.72 ± 0.40	*p* < 0.001^*∗∗*^
*p* ^†^	0.207	0.398	0.554	0.595	

^*∗*^Repeated measures ANOVA, *p* value for all visits. Post hoc analysis: statistically significant difference was observed from the preoperative visit to the 1-year visit (*p* < 0.001), from the 1-year visit to 3-year visit (*p*=0.036), and from 3-year visit to 5-year visit (*p*=001). ^*∗∗*^Repeated measures ANOVA, *p* value for all visits. Post hoc analysis: statistically significant difference was observed from the preoperative visit to the 1-year visit (*p* < 0.001), from the 1-year visit to 3-year visit (*p*=0.003), and from 3-year visit to 5-year visit (*p* < 0.001). ^†^Independent samples *t*-test.

**Table 3:** Refractive Sphere during follow-up.

	Preoperative (mean ± SD)	1 year (mean ± SD)	3 years (mean ± SD)	5 years (mean ± SD)	*p*
Veriflex	−11.21 ± 3.28	−0.10 ± 0.46	−0.24 ± 0.40	−0.41 ± 0.47	*p* < 0.001^*∗*^
Verisyse	−12.13 ± 3.34	−0.11 ± 0.39	−0.20 ± 0.43	−0.38 ± 0.46	*p* < 0.001^*∗∗*^
*p* ^†^	0.223	0.909	0.740	0.752	

^*∗*^Repeated measures ANOVA, *p* value for all visits. Post hoc analysis: statistically significant difference was observed from the preoperative visit to the 1-year visit (*p* < 0.001), from the 1-year visit to 3-year visit (*p*=0.026), and from 3-year visit to 5-year visit (*p* < 0.001). ^*∗∗*^Repeated measures ANOVA, *p* value for all visits. Post hoc analysis: statistically significant difference was observed from the preoperative visit to the 1-year visit (*p* < 0.001), from the 1-year visit to 3-year visit (*p*=0.010), and from 3-year visit to 5-year visit (*p* < 0.001). ^†^Independent samples *t*-test.

**Table 4:** Refractive cylinder during follow-up.

	Preoperative (mean ± SD)	1 year (mean ± SD)	3 years (mean ± SD)	5 years (mean ± SD)	*p* ^*∗*^
Veriflex	−0.59 ± 0.56	−0.52 ± 0.56	−0.51 ± 0.52	−0.54 ± 0.53	*p*=0.069
Verisyse	−0.75 ± 0.53	−0.64 ± 0.46	−0.68 ± 0.49	−0.70 ± 0.48	*p*=0.036^†^
*p* ^*∗∗*^	0.207	0.398	0.554	0.595	

^*∗*^Repeated measures ANOVA, *p* value for all visits. ^*∗∗*^Independent samples *t-*test. ^†^Post hoc analysis: statistically significant difference was observed from the preoperative visit to the 1-year visit (*p*=0.006) and from the preoperative visit to the 3-year visit (*p*=0.018). There was no statistically significant difference from the preoperative visit to the 5-year visit (*p*=0.162).

**Table 5:** Intraocular pressure during follow-up.

	Preoperative	1 year	3 years	5 years	*p* ^*∗*^
	Mean ± SD (range)	Mean ± SD (range)	Mean ± SD (range)	Mean ± SD (range)	
Veriflex	13.6 ± 1.7 (10 to 16)	13.9 ± 2.6 (10 to 21)	13.9 ± 2.1 (10 to 18)	13.5 ± 2.1 (10 to 21)	*p*=0.792
Verisyse	13.9 ± 2.0 (10 to 17)	13.9 ± 2.6 (9 to 20)	14.0 ± 1.5 (11 to 18)	14.3 ± 2.2 (10 to 20)	*p*=0.703
*p* ^*∗∗*^	0.561	0.950	0.825	0.088	

^*∗*^Repeated measures ANOVA, *p* value for all visits. ^*∗∗*^Independent samples *t*-test.

**Table 6:** Endothelial changes over the course of the study.

	Preoperative	1 year	3 years	5 years	*p* ^*∗*^
	*Central ECD (cells/mm^2^) (mean ± SD (% of cumulative ECD loss))*				
Verisyse	2681 ± 275 (N/A)	2599 ± 242 (3.05)	2534 ± 238 (5.48)	2482 ± 242 (7.42)	*p* < 0.001^†^
Veriflex	2656 ± 270 (N/A)	2575 ± 253 (3.04)	2512 ± 251 (5.42)	2460 ± 282 (7.64)	*p* < 0.001^††^
*p* ^*∗∗*^	0.692	0.670	0.678	0.709	

	*Coefficient of variation of cell area (%)*				
Verisyse	30.8 ± 5.5	29.0 ± 5.7	29.4 ± 4.3	29.1 ± 5.8	0.083
Veriflex	30.0 ± 5.2	30.6 ± 5.2	30.5 ± 5.4	31.2 ± 6.0	0.233
*p* ^†^	0.515	0.185	0.699	0.117	

	*Hexagonal cells (%)*				
Verisyse	65.4 ± 7.5	66.1 ± 7.3	64.8 ± 6.9	66.0 ± 8.2	0.114
Veriflex	62.7 ± 7.7	64.7 ± 8.4	63.9 ± 8.2	64.4 ± 8.8	0.142
*p* ^†^	0.130	0.422	0.601	0.396	

^*∗*^Repeated measures ANOVA, *p* value for all visits. ^*∗∗*^Independent samples *t*-test. ^†^Post hoc analysis: statistically significant difference was observed from the preoperative visit to the 1-year visit (*p* < 0.001), from the 1-year visit to 3-year visit (*p* < 0.001), and from 3-year visit to 5-year visit (*p* < 0.001). ^††^Post hoc analysis: statistically significant difference was observed from the preoperative visit to the 1-year visit (*p* < 0.001), from the 1-year visit to 3-year visit (*p* < 0.001), and from 3-year visit to 5-year visit (*p* < 0.001).

## Data Availability

The data used to support the findings of this study are available from the corresponding author upon request.
